# Hybrid Organic–Inorganic Perovskite Superstructures for Ultrapure Green Emissions

**DOI:** 10.3390/nano13050815

**Published:** 2023-02-22

**Authors:** Wen Kiat Chan, Jiawei Chen, Donglei Zhou, Junzhi Ye, Ricardo Javier Vázquez, Cheng Zhou, Guillermo Carlos Bazan, Akshay Rao, Zhongzheng Yu, Timothy Thatt Yang Tan

**Affiliations:** 1 School of Chemistry, Chemical Engineering and Biotechnology, Nanyang Technological University, Singapore 637459, Singapore; 2 Cavendish Laboratory, University of Cambridge, Cambridge CB3 0HE, UK; 3 State Key Laboratory of Integrated Optoelectronics, College of Electronic Science and Engineering, Jilin University, Changchun 130012, China; 4 Department of Chemistry, National University of Singapore, Singapore 117543, Singapore; 5 Institute for Functional Intelligent Materials, National University of Singapore, Singapore 117544, Singapore

**Keywords:** superstructures, organic–inorganic perovskites, ligand-assisted reprecipitation, ultrapure green emissions

## Abstract

All inorganic CsPbBr_3_ superstructures (SSs) have attracted much research interest due to their unique photophysical properties, such as their large emission red-shifts and super-radiant burst emissions. These properties are of particular interest in displays, lasers and photodetectors. Currently, the best-performing perovskite optoelectronic devices incorporate organic cations (methylammonium (MA), formamidinium (FA)), however, hybrid organic–inorganic perovskite SSs have not yet been investigated. This work is the first to report on the synthesis and photophysical characterization of APbBr_3_ (A = MA, FA, Cs) perovskite SSs using a facile ligand-assisted reprecipitation method. At higher concentrations, the hybrid organic–inorganic MA/FAPbBr_3_ nanocrystals self-assemble into SSs and produce red-shifted ultrapure green emissions, meeting the requirement of Rec. 2020 displays. We hope that this work will be seminal in advancing the exploration of perovskite SSs using mixed cation groups to further improve their optoelectronic applications.

## 1. Introduction

Lead halide perovskites with the general formula of APbX_3_ (A = Cs, methylammonium (MA), formamidinium (FA); X = Cl, Br, I) possess charge carriers with long diffusion lengths, which lead to mobile ionic defects and increase nonradiative recombination in perovskite bulk structures, limiting the performance of perovskite bulk structures [1,2]. Polavarapu’s group first explored the self-assembly of CsPbBr_3_ perovskite superstructures (SSs) as an option to overcome the shortcomings of perovskite bulk structures, proposing that the formation of SSs could reduce the probability of trap-assisted recombination by limiting the diffusion length of mobile carriers through electronically coupling individual high photoluminescence quantum yield (PLQY) nanocrystals (NCs) [3]. When SSs are formed, the electronic wavefunctions of the individual NCs overlap to form minibands in both valence and conduction bands, causing the interdot effect, which red-shifts the emission spectra of the SSs [3,4,5,6,7,8]. Upon dilution, the SSs would then break up into individual NCs, causing the emission spectra to be blue-shifted [3,9,10]. As a proof of concept, the CsPbBr_3_ SSs-based light-emitting diode (LED) fabricated by Polavarapu’s group in the same work displayed green emissions of higher purity than those of similar LEDs, due to the red-shifted emissions of the SSs. Additionally, CsPbBr_3_ SSs were reported to produce superfluorescent emissions, which have narrowed, coherent and ultrafast decay rates, potentially allowing enhanced energy transfer and absorption [4,11,12,13,14].

The existence of SSs in colloidal solutions was directly observed by Huang et al., using in situ spectroscopy. In their work, they discovered that CsPbBr_3_ NCs self-assemble into SSs in colloidal solutions after hot injection synthesis [10]. A few studies on the formation mechanism of SSs typically attributed SS formation to either dipole–dipole interactions amongst perovskite NCs or to the van der Waals forces between the ligands and perovskite NCs [3,6,10]. In this aspect, our group recently proposed that excess PbBr_2_ acts as a ‘glue’ which connects individual NCs to form SSs by occupying the Br vacancies of CsPbBr_3_ NCs to create CsPbBr_3_–PbBr_2_ linkages [9].

Perovskite SSs can find potential applications in areas such as LEDs, lasers, X-ray scintillators, nanoantennas, and quantum processors, as the close-packed assemblies in SSs allow better conductivity, red-shifted emissions, and carrier mobility [3,4,15,16,17]. Tong et al., fabricated CsPbBr_3_ SS LEDs with red-shifted emissions compared to CsPbBr_3_ NCs, obtaining pure green electroluminescence (EL) [3]. In another work, Zhou et al., developed superfluorescence-based CsPbBr_3_ quantum-dot superlattice microcavities which allow picosecond scale radiative times, demonstrating the potential for lasing and quantum processors [14].

To date, SS studies have focused on all-inorganic CsPbBr_3_, although organic cations such as MA and FA have useful properties. For instance, FA and MA cations possess faster rotation, leading to a greater orbital overlap and easier polaron formation, reducing nonradiative recombination and increasing carrier lifetimes [18,19,20,21]. Large polarons in hybrid perovskites have been reported to protect hybrid perovskite thin films from electronic dephasing, allowing better performance at room temperature [22]. Furthermore, some of the best perovskite solar cells today incorporate hybrid perovskites [23,24,25]. Given the obvious advantages of organic cations in perovskites, there is an urgent need to study hybrid perovskite SSs. Apart from the choice of A-site cation, each synthesis technique involves different synthesis conditions, organic ligands and reagent concentrations, which could affect spontaneous SS formation [9]. Thus far, the perovskites used in SS formation were synthesized using either the hot injection [4,10,14,26] or ultrasonication [3] techniques. Therefore, further studies of perovskite SSs involving a different synthesis method, such as the commonly used ligand-assisted reprecipitation (LARP), are highly desirable. 

Pure green emission plays an important role for an ultrawide color gamut in next-generation displays. At present, the International Telecommunication Union Recommendation BT 2020 (Rec. 2020) calls for ultrapure-green emitters to have emissions of 525–535 nm with full-width at half-maximum (FWHM) < 25 nm, however, to date, there is nothing commercially available that meets this standard [27,28,29,30]. Li’s group previously demonstrated that CsPbBr_3_–Cs_4_PbBr_6_:Na Type II structures are able to achieve FWHM < 23 nm while achieving 97% of Rec. 2020. However, their synthesis strategy requires the heating of up to 600 °C which would require large inputs of energy, especially when scaled up [29]. Zeng’s group managed to obtain 95% of Rec. 2020 by performing OA^+^-to-FA^+^ exchange to convert OA_2_PbBr_4_ into FAPbBr_3_ while attaining FWHM < 25 nm [30]. Although different approaches have been undertaken to derive ultrapure-green emitters, it is still unclear for now as to which approach will be the most feasible towards commercialization. Therefore, more studies should be performed to uncover new approaches to prepare ultrapure green emitters.

**Scheme 1:**
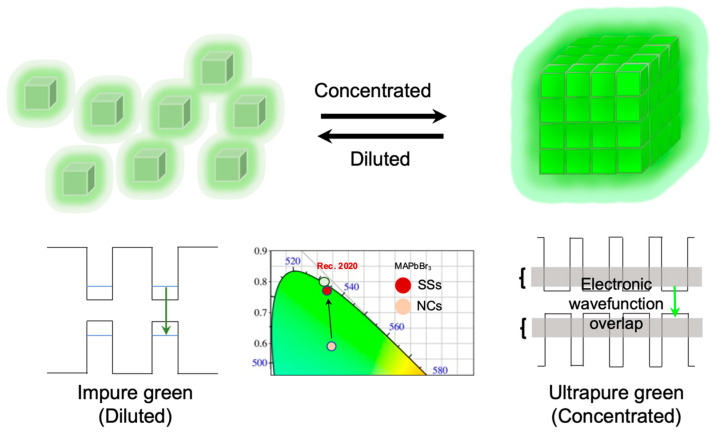
Perovskite NCs self-assemble into SSs at high concentrations but remain individual NCs at low concentrations. In SSs, the electronic wavefunctions overlap, leading to delocalized electrons and smaller band gaps than uncoupled NCs, causing red-shifted emissions. The red-shifted SSs emissions are ultrapure green suitable for the Rec. 2020 standard (red-shifted, shorter bright green arrow), while the NC emissions have lower green purity (blue-shifted, longer dark green arrow).

In this work, we demonstrate that hybrid perovskites (MAPbBr_3_ and FAPbBr_3_) and all-inorganic CsPbBr_3_ NCs synthesized through LARP could self-assemble into SSs both in a high-concentration colloidal solution and on thin film at high concentrations, but remain as individual NCs at low concentrations. When SSs are formed, the electronic wavefunctions of the individual NCs overlap and the interdot effect results lead to red-shifted emissions, allowing concentration-dependent emission tuning and longer recombination times [3,4,9,10]. For all SS samples, their emissions meet the Rec. 2020 standard for ultrapure green emissions (525–535 nm), whereas the NC emissions fell short of the standard (<520 nm) (Scheme 1) [27]. MAPbBr_3_ SS sample with the coordinate of (0.1770, 0.7718) shows the best match to the Rec. 2020 standard, achieving 96.2% of Rec. 2020. While the EL and light-emitting diode (LED) studies of these materials need to be further explored in future works, the ultrapure green emissions of the hybrid perovskite SSs underline the potential of these materials in developing efficient LED applications [31].

## 2. Materials and Methods

### 2.1. Materials

Methylammonium bromide (MABr, 98%), formamidinium bromide (FABr, ≥98%), cesium bromide (CsBr, 99.999% trace metals basis), lead bromide (PbBr_2_, 99.99%), *N*-*N*-dimethylformamide (DMF, suitable for HPLC, ≥99.9%), *n*-hexane (hexane, suitable for HPLC, ≥97.0%), toluene (suitable for HPLC, 99.9%), chloroform (≥99.5%), oleic acid (OA, 90%), oleylamine (OLA, 70%), and methyl acetate (anhydrous, 99.5%) were purchased from Sigma-Aldrich and used directly without further purification.

### 2.2. Synthesis

Colloidal MAPbBr_3_ NCs are synthesized based on a previously reported method with slight modifications [32,33]. Moreover, 0.0734 g of PbBr_2_, 0.0179 g of MABr, 50 µL of OLA and 500 µL of OA are co-dissolved sequentially by ultrasonication in 5 mL of DMF to form the precursor solution. Then, 10 mL of toluene is heated up on a hot plate to 55 °C with vigorous stirring, and 1 mL of the precursor solution is quickly injected into the heated toluene. A green mixture immediately forms, and the reaction mixture is cooled in an ice-water bath after 7 s. Methyl acetate (1:1 volume ratio) is added, and the mixture is then centrifuged at 10,000 rpm for 12 min. The precipitate is then dispersed in 2 mL of hexane and centrifuged at 7000 rpm for 5 min. The supernatant is kept for further analysis.

Colloidal FAPbBr_3_ NCs are synthesized based on a previously reported method with slight modifications [34]. Additionally, 0.0367 g of PbBr_2_, 0.0112 g of FABr, 40 µL of OLA, and 200 µL of OA are co-dissolved sequentially by ultrasonication in 1 mL of DMF to form the precursor solution. A total of 300 µL of the precursor solution is quickly injected into 10 mL of chloroform with vigorous stirring at room temperature. A green mixture immediately forms, and the reaction mixture is cooled in an ice-water bath after 7 s. Methyl acetate (1:1 volume ratio) is added, and the mixture is then centrifuged at 10,000 rpm for 12 min. The precipitate is then dispersed in 2 mL of hexane and centrifuged at 7000 rpm for 5 min. The supernatant is kept for further analysis.

Colloidal CsPbBr_3_ NCs are synthesized based on a previously reported method with slight modifications [35]. Moreover, 0.0734 g of PbBr_2_, 0.0426 g of CsBr, 200 µL of OLA, and 500 µL of OA are co-dissolved sequentially by ultrasonication in 5 mL of DMF to form the precursor solution. Then, 1 mL of the precursor solution is quickly injected into 10 mL of toluene with vigorous stirring at room temperature. A green mixture immediately forms, and the reaction mixture is cooled in an ice-water bath after 7 s. Methyl acetate (1:1 volume ratio) is added, and the mixture is then centrifuged at 10,000 rpm for 12 min. The precipitate is then dispersed in 2 mL of hexane and centrifuged at 7000 rpm for 5 min. The supernatant is kept for further analysis. 

Cs/MA/FAPbBr_3_ SSs naturally occur in colloidal solutions of higher concentrations [9,10]. Cs/MA/FAPbBr_3_ SSs thin films are prepared by drop-casting samples at higher concentrations and leaving the samples to dry in a fume hood at ambient temperature. Upon drop-casting, SSs spontaneously form without any need for annealing, regardless of substrate used. In TEM characterization, the samples are drop-cast onto copper grid. For PL analysis, APbBr_3_ NCs and SSs thin films are formed by drop-casting 0.2 mg mL^−1^ and 20 mg mL^−1^ samples, respectively, on ITO-coated glass. For SEM, the samples are drop-casted at concentrations of 0.2 mg mL^−1^ (for NCs) and 20 mg mL^−1^ (for SSs) onto a carbon tape substrate, while for AFM, the samples are drop-casted onto microscope glass.

### 2.3. Characterization

Transmission electron microscopy (TEM), high-resolution TEM (HRTEM), dark field-scanning TEM (DF-TEM), and energy-dispersive X-ray spectroscopy (EDS) images were captured using a JEOL TEM-2100F transmission electron microscope operating at 200 kV. The length of the NCs for all the samples were measured using ImageJ software, with 100 NCs measured for each sample. X-ray diffraction (XRD) was conducted with a D8 Advance Bruker X-ray diffractometer with Cu Kα radiation (λ = 1.5406 Å) from 10 ° to 50° with a counting time of 0.5 s per step. Scanning electron microscopy (SEM) micrographs were captured using a JEOL JSM-6390LA scanning electron microscope. Atomic force microscopy (AFM) images were taken with a ScanAsyst-Otespa probe on a Bruker NanoScope V atomic force microscope. 

The Debye-Scherrer crystallite size was calculated using the equation: Crystalline size, D = kλ/βcosθ, where k = 0.94, λ = 0.15406 nm, β = FWHM of the diffraction peak, and θ is the diffraction angle.

UV–Vis absorption spectra were obtained using a Shimadzu UV3600 Spectrometer by dispersing the samples in hexane at various concentrations in a standard quartz cuvette at room temperature. PL spectra in the visible range were obtained using a Fluoromax-4, Horiba Jobin Yvon Spectrofluorometer by dispersing the samples in hexane at various concentrations in a standard quartz cuvette (for colloidal samples) or drop-casting samples onto ITO glass at room temperature (for thin film samples), with a 150 W continuous wavelength Xenon arc lamp as the excitation source and broadband optical power at focal point of 15 W. The PLQY measurements of the thin film samples were conducted using the Fluoromax-4, Horiba Jobin Yvon Spectrofluorometer equipped with the same 150 W continuous wavelength Xenon arc lamp as the excitation source together with an integrating sphere. The PL lifetime measurements were obtained at room temperature on a Fluorolog photoluminescence spectrometer from Horiba Scientific equipped with a DeltaDiode 402 nm laser (DD-510L), having an average power of 1.4 mW, a peak power of 100 mW, and a pulse width of 110 ps. 

The fitting of lifetimes is based on the mono-exponential decay function for Rhodamine B:

y=y0+A1e−x−x0t1

The bi-exponential decay function is used to fit the lifetime of APbBr_3_:

y=y0+A1e−x−x0t1+A2e−x−x0t2

$$\tau_{average}=\frac{\left(A_{1}\times t_{1}^{2}+A_{2}\times t_{2}^{2}\right)}{A_{1}\times t_{1}+A_{2}\times t_{2}}$$

## 3. Results and Discussion

**Figure 1:**
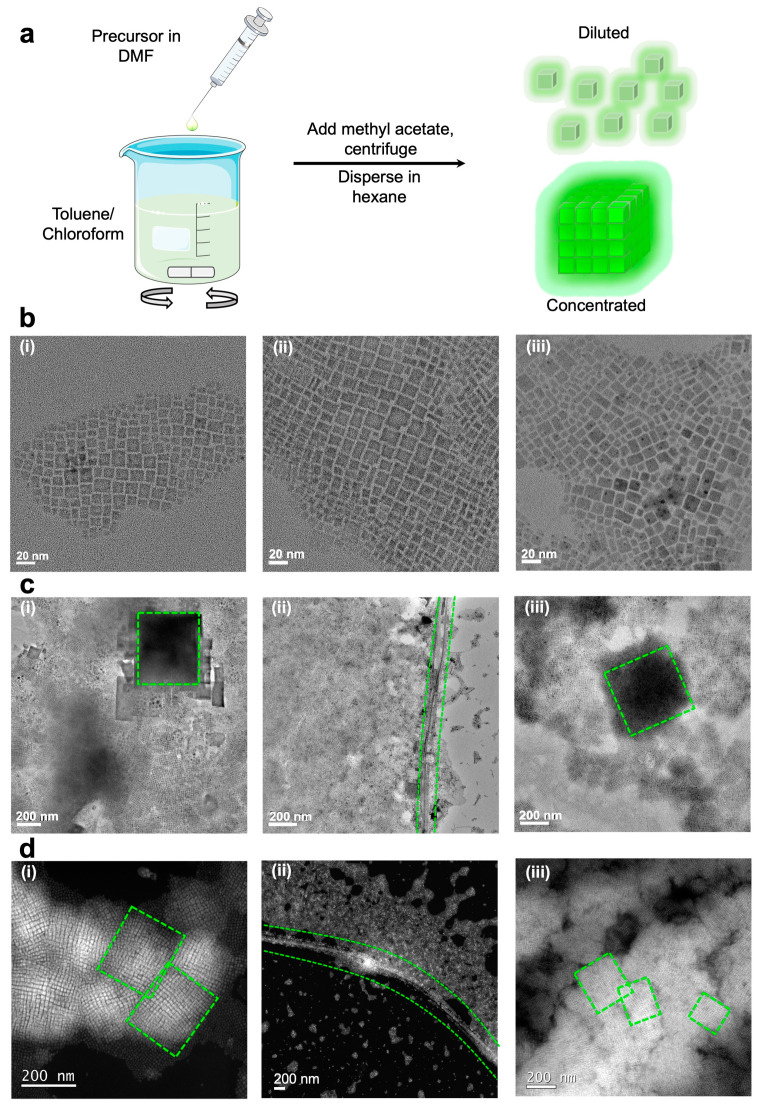
(**a**) Schematic illustration of the LARP synthesis process. (**b**) TEM images of (**i**) MAPbBr_3_, (**ii**) FAPbBr_3_, and (**iii**) CsPbBr_3_ NCs. (**c**) TEM images of (**i**) MAPbBr_3_, (**ii**) FAPbBr_3_, and (**iii**) CsPbBr_3_ SSs. (**d**) DF-STEM images of (**i**) MAPbBr_3_, (**ii**) FAPbBr_3_, and (**iii**) CsPbBr_3_ SSs, with the location of SSs highlighted within the green lines/boxes.

MAPbBr_3_, FAPbBr_3_, and CsPbBr_3_ NCs were first synthesized by dissolving the required precursors in dimethylformamide (DMF), which acts as a ‘good’ solvent, and injecting into excess amounts of ‘bad’ solvents such as toluene and chloroform (See Section 2) [32,33,34,35]. The perovskite NCs were formed within seconds and were subsequently washed with methyl acetate before dispersing in hexane for further analysis (Figure 1a). We then analyzed the morphology of the samples prepared at different perovskite concentrations (5 and 20 mg mL^−1^) using transmission electron microscopy (TEM). The samples are drop-casted onto copper grids at different concentrations and left to dry in a fume hood under ambient conditions to form a thin film. For samples prepared at 5 mg mL^−1^, the TEM images revealed rectangular MAPbBr_3_, FAPbBr_3_, and CsPbBr_3_ NCs of sizes 9.9 ± 2.0 nm, 10.8 ± 2.9 nm, and 10.0 ± 2.6 nm, respectively (Figure 1b and Figure S1). The TEM (Figure 1c) and dark-field scanning TEM (DF-STEM) (Figure 1d and Figure S2) images of the same samples prepared at 20 mg mL^−1^ confirm the formation of SSs, which were not observed at 5 mg mL^−1^. MAPbBr_3_ and CsPbBr_3_ SSs form rectangular-shaped SSs of sizes > 200 nm, but FAPbBr_3_ interestingly forms NWs with a thickness of < 100 nm but of length > 1 μm. Given that the ligand system used is the same for all three samples (oleic acid (OA) and oleylamine (OLA)), the NC sizes and shapes are similar, and that all samples were dispersed in hexane, the difference in SS morphology is most likely attributed to the different sizes of the A-site cations. FA^+^ has a larger ionic radius (0.253 nm) than MA^+^ (0.217 nm) and Cs^+^ (0.188 nm), and as such, FAPbBr_3_ has a comparably larger crystal lattice than MAPbBr_3_ and CsPbBr_3_, which could potentially favor the formation of NW SSs [36].

**Figure 2:**
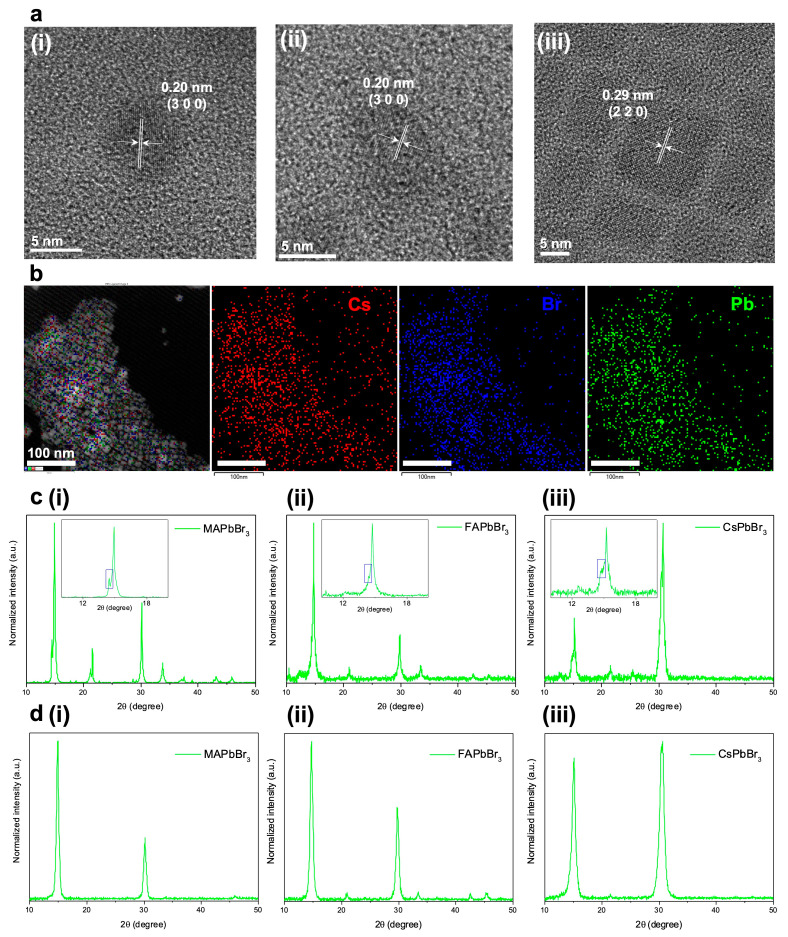
(**a**) HRTEM images of (**i**) MAPbBr_3_, (**ii**) FAPbBr_3_, and (**iii**) CsPbBr_3_ NCs, respectively. (**b**) EDS scan of CsPbBr_3_ NCs. (**c**) XRD patterns of SSs of (**i**) MAPbBr_3_, (**ii**) FAPbBr_3_, and (**iii**) CsPbBr_3_, with insets emphasizing satellite peaks resulting from reflection splitting due to SS formation. (**d**) XRD patterns of NCs of (**i**) MAPbBr_3_, (**ii**) FAPbBr_3_, and (**iii**) CsPbBr_3_.

High-resolution TEM (HRTEM) images of the samples were obtained, and the lattice distances measured were consistent with those of MAPbBr_3_, FAPbBr_3_, and CsPbBr_3_ (Figure 2a). The energy-dispersive spectroscopy (EDS) mapping was performed on CsPbBr_3_, which confirms the successful formation of CsPbBr_3_ (Figure 2b). X-ray diffraction (XRD) patterns were obtained for both SSs (concentrated drop-cast) and NCs (diluted drop-cast) and it is confirmed that pure cubic phase MAPbBr_3_, FAPbBr_3_, and CsPbBr_3_ were synthesized. The main difference between the XRD patterns of SSs and NCs is the presence of split reflections into satellite peaks at approximately 2θ = 15° in the XRD pattern of SSs (Figure 2c) compared to the absence of satellite peaks for the NC XRD patterns (Figure 2d) [37,38,39]. The importance of the satellite peaks in determining the presence of SSs was explored by Manna’s group, where they studied the XRD patterns of self-assembled CsPbBr_3_ SSs prepared by depositing the concentrated CsPbBr_3_ NC solution onto thin films and discovered that satellite peaks form at approximately 2θ = 15°. NCs packed at high concentrations self-assemble into SSs, leading to the precise spatial periodicity of NCs. In turn, this spatial periodicity causes the interference of the X-rays used in XRD analysis leading to the split peaks which were observed [39]. Therefore, the presence of the XRD split peaks further confirms that SSs were formed for our samples under high NC concentrations. Apart from the split peaks, the FWHM of the XRD patterns are narrowed for the SSs compared to their corresponding NCs, implying larger Scherrer–Debye average crystallite sizes for the SS samples. The Scherrer–Debye average crystallite sizes for the MAPbBr_3_, FAPbBr_3_, and CsPbBr_3_ SSs are 29.0 nm (14.9 nm for NC), 23.9 nm (16.7 nm for NC), and 15.6 nm (9.1 nm for NC), respectively, and are consistently higher than their corresponding NCs.

**Figure 3:**
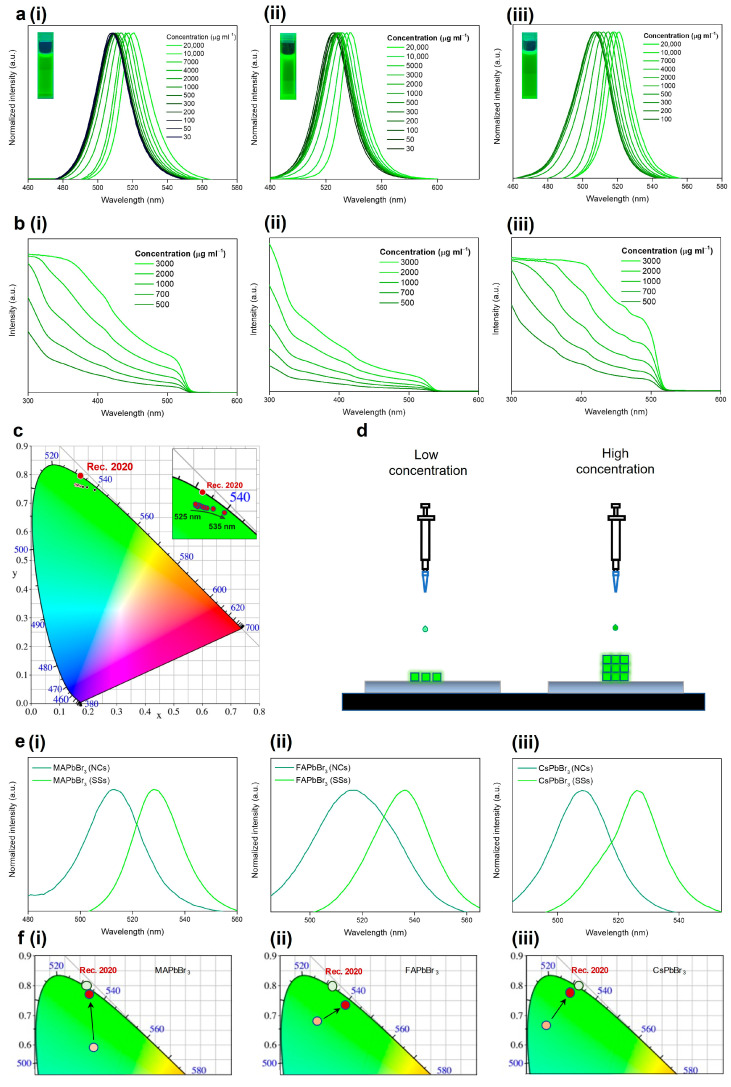
(**a**) PL spectra of the colloidal (**i**) MAPbBr_3_, (**ii**) FAPbBr_3_, and (**iii**) CsPbBr_3_ under 365 nm excitation. (**b**) Absorption spectra of (**i**) MAPbBr_3_, (**ii**) FAPbBr_3_, and (**iii**) CsPbBr_3_. All colloidal samples were dispersed in hexane. (**c**) Color gamut in the CIE chromaticity diagram of colloidal MAPbBr_3_ at different concentrations. (**d**) Schematic illustration of sample preparation on ITO film. (**e**) PL spectra and (**f**) CIE chromaticity diagrams of (**e,f**(**i**)) MAPbBr_3_, (**e,f**(**ii**)) FAPbBr_3_, and (**e,f**(**iii**)) CsPbBr_3_ thin-film NCs (light red) and SSs (dark red) emissions under 365 nm excitation.

We then compare the PL and absorption properties of the respective samples in the colloidal form and when dispersed on thin film at different concentrations. For samples in colloidal form, all three samples are bright green under UV light, and the emission peaks are red-shifted at higher concentrations due to either the reabsorption or the interdot effect, while the emission peaks are blue-shifted upon dilution (Figure 3a) [3,4,9,10,40,41]. While it has been initially debated that reabsorption between perovskite NCs could cause concentration-dependent red-shifted, narrowed emissions [3,42], we rule out reabsorption as the major factor affecting perovskite SS emission. This is because there is a growing body of evidence proposing that the red-shifted emissions arise from electronic coupling. Rainò et al., Tong et al., and Zhou et al., in separate studies, assembled CsPbBr_3_ SSs and obtained narrowed, red-shifted emissions that they found to be due to electron coupling which was absent in isolated NCs [3,4,14]. More recently, Schall’s group reported the synthesis of perovskite superballs consisting of spherical SSs, where they found electronic coupling which they believe could be further improved by replacing the ligands to shorter, more conductive ones [8]. Electronic coupling and the interdot effect from miniband formation was also reported for other material systems such as perovskite-type superlattices and InGaAs quantum dots [5,43]. As perovskite NCs are known to spontaneously form SSs at higher colloidal concentrations, the red-shifted emissions are thus attributed to the SSs in the colloidal solutions. Furthermore, based on our concentration-dependent emission spectra, the red-shift happens more significantly as the concentration increases and this red-shift slows down at a higher concentration, implying that electronic coupling is stronger with the nearest neighboring NCs, consistent with interdot coupling rather than reabsorption [3].

While the emission peaks change with concentration, the absorption band edges of the samples are constant and do not change with concentration, which is also consistent with previous works (Figure 3b) [9,10]. Notably, the Rec. 2020 standard requires a narrow range of 525–535 nm with FWHM < 25 nm for ultrapure green emission. The fine-tuning of hybrid organic–inorganic perovskite concentrations in colloidal form could meet the Rec. 2020 standard as both the emission ranges and FWHMs of these organic–inorganic perovskite samples could fall in the proper range, while the pure inorganic CsPbBr_3_ would not reach above 521 nm (Figure 3a and Tables S1–S3). For FAPbBr_3_, the requirement for FWHM < 25 nm can only be achieved at higher concentrations above 0.3 mg mL^−1^. For MAPbBr_3_, the Commission Internationale de l’Eclairage (CIE) coordinates could also be finely tuned from (0.1546, 0.7673) to (0.2495, 0.7301) in colloidal form with emission wavelengths falling within 525–535 nm, allowing the emissions to approach the Rec. 2020 standard, similarly to previous works involving emission tuning (Figure 3c) [3,44,45].

Although FAPbBr_3_ and MAPbBr_3_ at high concentrations have shown promise in obtaining a pure green light, perovskite SSs on thin films may have further red-shifted emissions towards pure green light and closer to the application scenarios. We therefore now focus on the PL properties of our APbBr_3_ samples when dispersed on thin films. From the TEM and DF-STEM images, we observed that MAPbBr_3_, FAPbBr_3_, and CsPbBr_3_ all form SSs in the thin films when prepared under high sample concentration and remain as individual NCs at low concentrations. The three samples were drop casted at both a high concentration (20 mg mL^−1^) and low concentration (0.2 mg mL^−1^) onto ITO-coated glass to prepare the thin film samples of SSs and NCs, respectively (Figure 3d). Under 365 nm excitation, MAPbBr_3_ emits at 512 nm (NCs) and 528 nm (SSs), FAPbBr_3_ emits at 516 nm (NCs) and 536 nm (SSs), while CsPbBr_3_ emits at 508 nm (NCs) and 526 nm (SSs). 

In all cases, the SS emission is red-shifted compared to the NC emission due to the interdot effect (Figure 3e) [3,4,9,10,27]. The FWHMs of SS films have been narrowed from 26 to 22 nm (MAPbBr_3_), 39 to 26 nm (FAPbBr_3_), and 24 to 21 nm (CsPbBr_3_), respectively, compared to NC films. All the SS films show a significant move towards to Rec. 2020 standard compared to NC films, as shown in the CIE diagrams (Figure 3f). The MAPbBr_3_ SS sample with the coordinate of (0.1770, 0.7718) shows the best match to the Rec. 2020 standard of ultrapure green light (0.170, 0.797), achieving 96.2% of Rec. 2020. To the best of our knowledge, the MAPbBr_3_ SSs represent one of the “greenest” emissions ever reported in perovskite-based systems.

**Figure 4:**
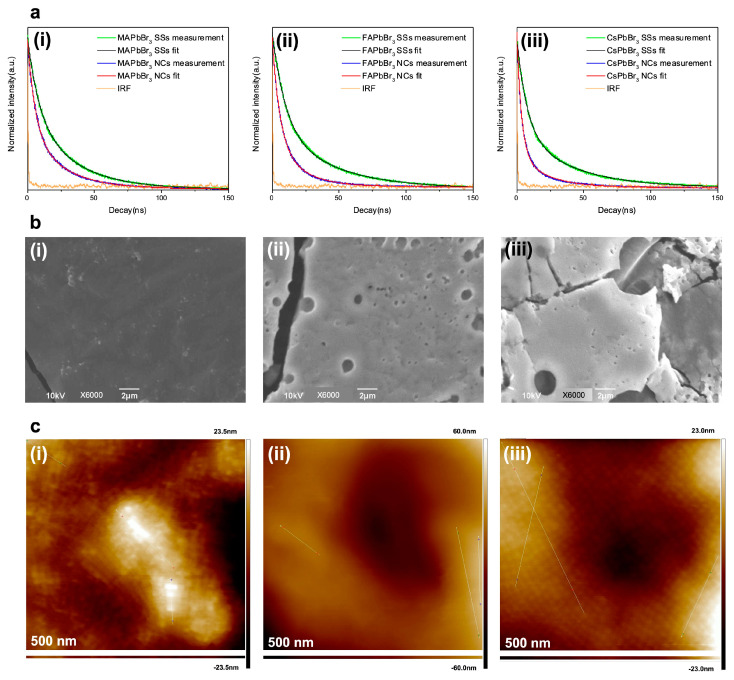
PL lifetime profiles of (**a**) (**i**) MAPbBr_3_, (**ii**) FAPbBr_3_, and (**iii**) CsPbBr_3_ SSs and NCs. The excitation wavelength was 402 nm. The detection wavelength was 515 nm for MAPbBr_3_ and CsPbBr_3_ and 517 nm for FAPbBr_3_. The SEM images of (**b**) (**i**) MAPbBr_3_, (**ii**) FAPbBr_3_, and (**iii**) CsPbBr_3_ SSs. And AFM height images of (**c**) (**i**) MAPbBr_3_, (**ii**) FAPbBr_3_, and (**iii**) CsPbBr_3_ SSs.

The time-correlated single-photon counting (TCSPC) technique was used to probe the PL lifetimes of the investigated systems and gain further insights into the energy transfer mechanism of SSs compared to NCs. Samples were drop-casted on ITO-coated glasses. The experiments were conducted at ambient temperature with a 402 nm excitation source, and the well-known Rhodamine B was used as the standard. A mono-exponential decay lifetime of 2.5 ns was obtained for Rhodamine B, which is longer than the instrument response function (IRF) and agreed with literature precedents (Figure S3 and Table S4) [46,47]. The PL lifetime profiles of MAPbBr_3_, FAPbBr_3_, and CsPbBr_3_ are then measured and fitted with the bi-exponential decay function (R^2^ = 0.99 for all three samples) (Figure 4a and Tables S5–S7). In all cases, we observe a lengthening in the fluorescence lifetime (τ_average_) of the SSs relative to their NCs counterparts form (Table 1). In the case of MAPbBr_3_, the PL lifetime increases from 20.8 ns to 27.3 ns, while the PL lifetimes of FAPbBr_3_ and CsPbBr_3_ increase from 14.9 ns to 34.4 ns and from 15.1 ns to 31.7 ns, respectively. These results revealed 1.3, 2.3, and 2.1-fold emissive lifetime increments in MAPbBr_3_, FAPbBr_3_, and CsPbBr_3_ SSs compared to their NCs form. Adapting an approach from Perumal et al., we now analyze the contributions of the slower and faster components of the PL lifetime. For MAPbBr_3_, the faster component contributes 64% (36% for slower component) to NCs compared to 47% for SSs. The faster component contribution is also reduced from 70% (NCs) to 60% (SSs) for FAPbBr_3_ and from 81% (NCs) to 62% (SSs) in the case of CsPbBr_3_. These data imply that when SSs are formed, the slower component is increasingly favored due to electron delocalization [48]. When SSs are formed, the electronic wavefunctions of the constituent NCs overlap with each other, leading to enhanced electronic coupling and the interdot effect, which could cause the increased lifetimes of SSs compared to NCs [10]. Furthermore, SSs consists of close-packed NCs which results in larger dielectric constants which provides stronger Coulombic interaction. This enhanced screening reduces the exciton binding energy and thus slows down the recombination process, contributing to the increased lifetimes of SSs [49]. In a previous study, Kim et al., measured the PL lifetimes of CsPbBr_3_ at high and low concentrations and found that the NCs increasing the concentration leads to a longer lifetime, which is consistent with our findings. They proposed that, when the distances between NCs are smaller, the probability of non-radiative energy transfer between the NCs occur leading to longer lifetimes for the concentration solutions. While the term interdot effect was not used, their explanations mirror that of the interdot effect in terms of electron decentralization [50]. Besides forming SSs, the PL lifetimes of perovskites could be further increased using mixed cations and anions at the perovskite A- and X-sites. Mazanik’s group discovered that the PL lifetime increased by 2.4 times for (Cs/MA/FA)Pb(I/Br)_3_ and 1.8 times for (Cs/FA)Pb(I/Br)_3_ when compared to MAPbI_3_ due to the electron diffusivity change when the localizing states are modified by the types of constituent ions. This implies room for future work where the SSs of mixed A-site cations could be developed and explored [51]. In addition, we measured the PLQYs of SSs on thin films prepared by drop-casting to examine whether the formation of SSs affect the PLQYs under 365 nm excitation. The PLQYs of MAPbBr_3_, FAPbBr_3_, and CsPbBr_3_ NCs on thin films are 17%, 10%, and 7%, while the PLQYs of their corresponding SSs on thin films are 12%, 11%, and 7%, respectively. This supports the finding of a previous work which reported that SS formation does not severely impact the PLQY of perovskite NCs and could potentially even enhance the PLQY [3]. The presence of ferroelectric domains has been proposed to be a key factor affecting optical performance, as it could aid the separation of photoexcited electron and hole pairs and reduce the electron and hole recombination, thus improving the optical performance. The presence of dipoles in the A-site cation of perovskites gives rise to ferroelectric domains due to the lone pair of electrons in the Pb^2+^ ions of the perovskite cage being prone to structural distortions. The dipoles in the Debye of MA^+^ (2.29) is much larger than that of both FA^+^ (0.21) and Cs^+^ (0) and thus the ferroelectric domains affect the optical performance of MAPbBr_3_ more than FA/CsPbBr_3_. Therefore, it is possible that the formation of SSs which leads to electron delocalization could negate the positive effects of these ferroelectric domains, leading to a decrease in PLQY for MAPbBr_3_ SSs compared to NCs [52,53]. The measurement of PLQY of colloidal SSs is very difficult as the concentration is too high and any dilution will cause the SSs to break apart. This issue was previously highlighted by Huang et al., in their study of colloidal CsPbBr_3_ SSs [10], and the PLQY studies in this work are therefore limited to thin film samples.

Scanning electron microscopy (SEM) images and atomic force microscopy (AFM) height images of the drop-casted samples on the thin film were captured to better understand the film structure and macroscopic properties. The SEM images of the samples prepared at higher (SS) concentrations (Figure 4b) reveal a uniform-looking film with some oval empty spots due to solvent evaporation, while the SEM images for the samples prepared at lower concentrations show individual clusters of NCs (Figure S4a). This is consistent with our TEM observation where the NCs tend to form clusters when close together rather than individually separated. The height and roughness analysis of the SS films (Figure 4c) and NC clusters (Figure S4b) were then conducted based on the AFM height images. The section analysis of the samples indicates that the thickness and roughness of both SS films and their corresponding NC clusters are similar (Table S8). This further confirms that NCs are naturally inclined to self-assemble such that when the sample concentration is low, the NCs clump together to form the clusters of thickness comparable of SS films [15]. Due to the nature of drop-casting, the films are radial in dimension with the samples clustered more at the center. The maximum thickness at the center position for the SS films (MAPbBr_3_: 109 nm, FAPbBr_3_: 88 nm, and CsPbBr_3_ SSs: 63 nm) is consistently higher than that of their corresponding NC films (MAPbBr_3_: 20 nm; FAPbBr_3_: 20 nm, and CsPbBr_3_: 17 nm) which is expected given the higher concentration used to prepare the SS films.

## 4. Conclusions

In conclusion, we demonstrated that hybrid perovskites such as MAPbBr_3_ and FAPbBr_3_ and all-inorganic CsPbBr_3_ synthesized through the facile LARP technique could also self-assemble into SSs when drop casted onto ITO-coated glass. We showed that in all cases, APbBr_3_ perovskite SSs have red-shifted emissions compared to their NCs counterparts, providing a method of emission tuning to obtain ultrapure green emissions by changing perovskite concentration. We finally studied the PL lifetime profiles of APbBr_3_ and found that, in all cases, APbBr_3_ have longer lifetimes as SSs compared to their corresponding APbBr_3_ NCs. When SSs are formed, the individual NCs become electronically coupled, leading to the delocalization of electrons and longer recombination times, potentially improving the optoelectronic performance. At lower concentrations, electronically isolated NCs are formed, which results in shorter PL lifetimes and blue-shifted emissions. The ultrapure green emissions from the hybrid perovskite SSs demonstrate its potential for further exploration in LED device fabrication and optoelectronic applications. As the drop-casting method could lead to inhomogeneous thin films, follow-up studies should be performed using blade-coating, spin-coating, or drying-mediated self-assembly techniques which will lead to more uniform thin films. The study of perovskite SSs is still in the early stages, and we hope that this work will lead to the further development of hybrid and mixed-cation SSs involving other A-site cations such as guanidinium, Rb^+^ and K^+^, to further enhance the optical performance of perovskite solar cells, LEDs, lasers, and photodetectors. Furthermore, the SSs of doped and surface-passivated perovskites could also be explored to obtain superior optical performance [54]. 

## Figures and Tables

**Table 1:** Average fluorescence lifetime τ_average_.

Sample	SSs τ_average_ (ns)	NCs τ_average_ (ns)	Enhancement Factor $\frac{\tau_{average\;SC}}{\tau_{average\;NC}}$
MAPbBr_3_	27.3	20.8	1.3
FAPbBr_3_	34.4	14.9	2.3
CsPbBr_3_	31.7	15.1	2.1

## Data Availability

Data available upon written request to corresponding author.

## Supplementary Materials

The following supporting information can be downloaded at: https://www.mdpi.com/article/10.3390/nano13050815/s1. Figure S1: Particle size distributions of APbBr_3_ NCs; Figure S2: DF-STEM images of FAPbBr_3_ and CsPbBr_3_ SSs. Figure S3: PL decay profile of Rhodamine B; Table S1: Optical performance of MAPbBr_3_ SSs; Table S2: Optical performance of FAPbBr_3_ SSs; Table S3: Optical performance of CsPbBr_3_ SSs; Table S4: Fitting parameters for Rhodamine B; Table S5: Fitting parameters for MAPbBr_3_; Table S6: Fitting parameters for FAPbBr_3_; Table S7: Fitting parameters for CsPbBr_3_; Figure S4: SEM micrographs and AFM height images of NCs; Table S8: Section analysis of AFM height images.
