# Supplementary material for: Hybrid Organic–Inorganic Perovskite Superstructures for Ultrapure Green Emissions
Source: Nanomaterials (Basel). 2023 Feb 22;13(5):815. doi: 10.3390/nano13050815 (PMC10005548; doi:10.3390/nano13050815)
Supplement: Supplementary file 1 [file nanomaterials-13-00815-s001.zip › nanomaterials-2183101-supplementary.pdf]

nanomaterials

Supplementary Materials

Hybrid Organic–Inorganic Perovskite Superstructures for Ultrapure Green Emissions

Wen Kiat Chan [1], Jiawei Chen [2], Donglei Zhou [3], Junzhi Ye [2], Ricardo Javier Vázquez [4,5], Cheng Zhou [4], Guillermo Carlos Bazan [4,5], Akshay Rao [2], Zhongzheng Yu [1,2,*] and Timothy Thatt Yang Tan [1,*]

1 School of Chemistry, Chemical Engineering and Biotechnology, Nanyang Technological University, Singapore 637459, Singapore
2 Cavendish Laboratory, University of Cambridge, Cambridge CB3 0HE, UK
3 State Key Laboratory of Integrated Optoelectronics, College of Electronic Science and Engineering, Jilin University, Changchun 130012, China
4 Department of Chemistry, National University of Singapore, Singapore 117543, Singapore
5 Institute for Functional Intelligent Materials, National University of Singapore, Singapore 117544, Singapore
* Correspondence: yuzh0010@e.ntu.edu.sg (Z.Y.); tytan@ntu.edu.sg.com (T.T.Y.T.)

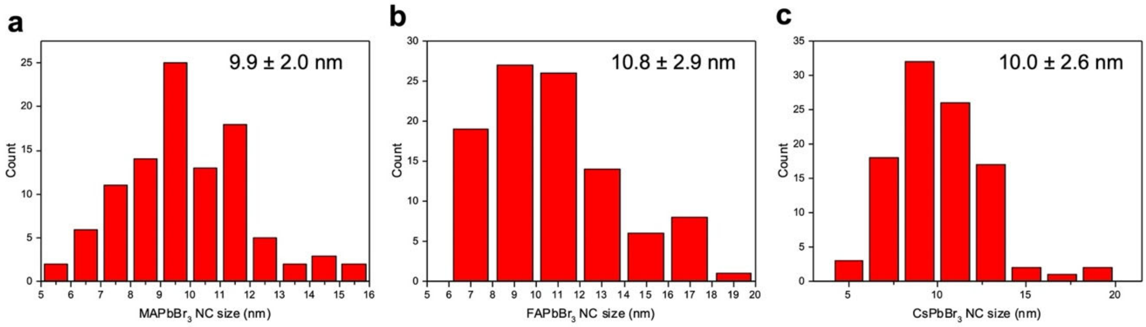

Figure S1. Particle size distributions of (**a**) $MAPbBr_3$, (**b**) $FAPbBr_3$, and (**c**) $CsPbBr_3$ NCs.

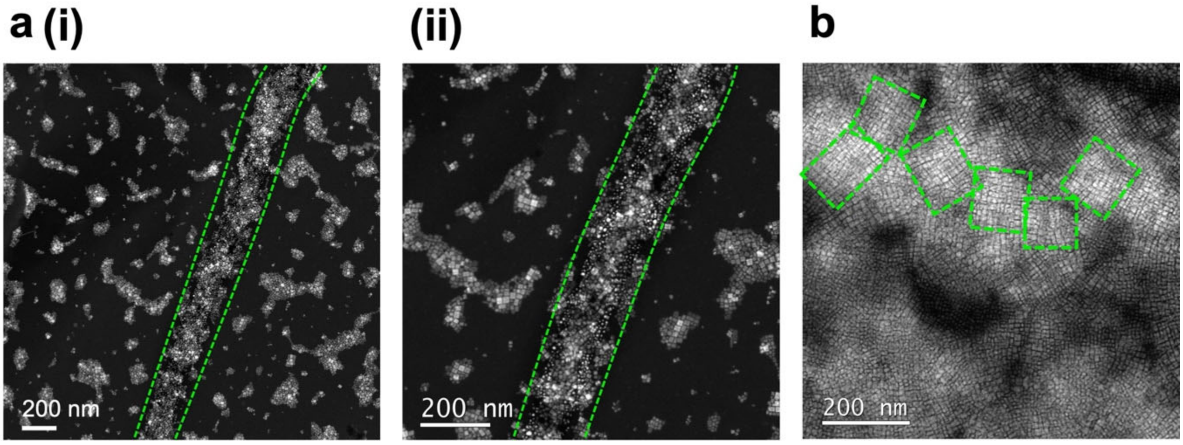

Figure S2. DF-STEM images of (**a**) $FAPbBr_3$ SS, and **(b)** $CsPbBr_3$ SSs, with green lines/boxes emphasizing location of SSs. (i) and (ii) show the same $FAPbBr_3$ SS at different magnifications.

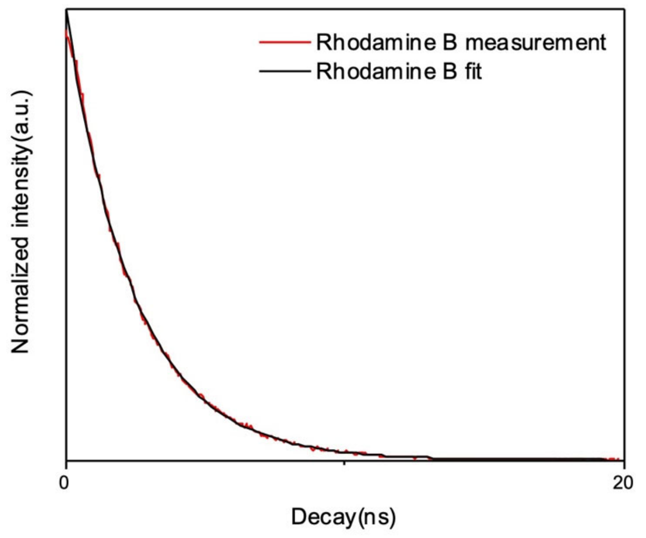

Figure S3. PL decay profile of Rhodamine B was used as the standard reference.

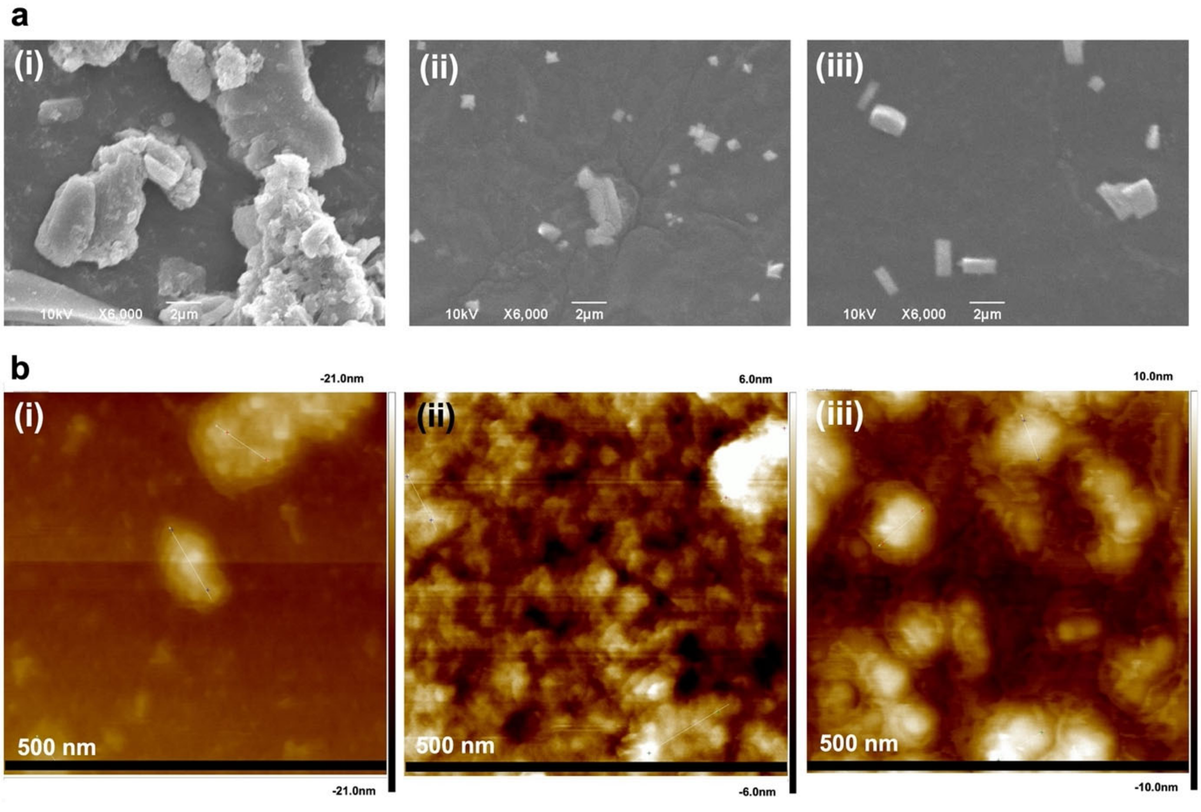

Figure S4. SEM micrographs of (**a**)(**i**) $MAPbBr_3$, (**ii**) $FAPbBr_3$, and (**iii**) $CsPbBr_3$ NCs. AFM height images of (**b**)(**i**) $MAPbBr_3$, (**ii**) $FAPbBr_3$, and (**iii**) $CsPbBr_3$ NCs.

Table S1. Optical performance of $MAPbBr_3$ colloidal solution.

Concentration (μg mL^{-1})	CIE X	CIE Y	Peak (nm)	FWHM (nm)
30	0.0666	0.6692	508	22
50	0.0657	0.6676	509	23
100	0.067	0.6767	508	22
200	0.0681	0.6811	509	21
300	0.0693	0.6819	509	23

500	0.0697	0.6865	510	22
1000	0.0732	0.6944	510	22
2000	0.0764	0.6937	511	21
4000	0.0858	0.7347	513	20
7000	0.1019	0.7688	516	19
10,000	0.1129	0.7743	518	19
20,000	0.1355	0.7776	521	20

Table S2. Optical performance of $FAPbBr_3$ colloidal solution.

Concentration (μg mL^{-1})	CIE X	CIE Y	Peak (nm)	FWHM (nm)
30	0.1546	0.7673	525	25
50	0.1559	0.7624	526	27
100	0.1606	0.7609	527	27
200	0.162	0.7634	527	26
300	0.1656	0.7624	527	26
500	0.171	0.7601	528	26
1000	0.1724	0.76	529	26
2000	0.1754	0.7587	529	26
3000	0.1808	0.7576	530	25
5000	0.196	0.7554	531	24
10,000	0.2226	0.7469	535	21
20,000	0.2495	0.7301	537	20

Table S3. Optical performance of $CsPbBr_3$ colloidal solution.

Concentration (μg mL^{-1})	CIE X	CIE Y	Peak (nm)	FWHM (nm)
100	0.0507	0.5919	507	24
200	0.0521	0.5863	507	25
300	0.0518	0.5949	507	24
500	0.0534	0.6086	508	24
1000	0.0557	0.6499	509	22
2000	0.0623	0.7188	512	19
4000	0.076	0.7665	515	17
7000	0.0941	0.7853	517	16
10,000	0.1038	0.7887	518	16
20,000	0.1183	0.7889	521	16

Table S4. Fitting parameters for Rhodamine B.

**Equation:** $\boldsymbol{y = y_0 + A_1 e^{-(x-x_0)/t_1}}$	
$\boldsymbol{y_0}$	3.47417 × 10^{-4}
A_1	1.04531
x_0	0.00641
t_1	2.46078

Table S5. Fitting parameters for $MAPbBr_3$.

Equation: $y = y_0 + A_1 e^{-(x-x_0)/t_1} + A_2 e^{-(x-x_0)/t_2}$		
	**SSs**	NCs
y_0	0.00355	0.00382
x_0	0.23568	−0.76354
A_1	0.45082	0.68597
t_1	8.16115	5.80553
A_2	0.51815	0.38134
t_2	31.59794	26.68215
$\tau_{average}$ (ns)	27.3	20.8

Table S6. Fitting parameters for $FAPbBr_3$.

Equation: $y = y_0 + A_1 e^{-(x-x_0)/t_1} + A_2 e^{-(x-x_0)/t_2}$		
	**SSs**	NCs
y_0	0.00629	0.01865
x_0	−0.41117	−0.32434
A_1	0.6326	0.7142
t_1	9.72035	5.67985
A_2	0.41514	0.30154
t_2	42.87193	20.86572
$\tau_{average}$ (ns)	34.4	14.9

Table S7. Fitting parameters for $CsPbBr_3$.

Equation: $y = y_0 + A_1 e^{-(x-x_0)/t_1} + A_2 e^{-(x-x_0)/t_2}$		
	**SSs**	NCs
y_0	0.0137	0.01234

x_0	−0.52144	−0.6776
A_1	0.63601	0.96134
t_1	7.39563	3.5217
A_2	0.38505	0.22251
t_2	39.21957	22.76281
$\tau_{average}$ (ns)	31.7	15.1

Table S8. Section analysis of AFM images.

**Sample**	**Thickness (nm)**	**Roughness (nm)**
$MAPbBr_3$	SS film: 5.82<br>NC cluster: 4.64	SS film: 1.81<br>NC cluster: 1.07
$FAPbBr_3$	SS film: 1.88<br>NC cluster: 2.36	SS film: 0.68<br>NC cluster: 0.75
$CsPbBr_3$	SS film: 2.75<br>NC cluster: 2.94	SS film: 0.942<br>NC cluster: 0.789
